# The regression trap: why regression analyses are not suitable for selecting determinants to target in behavior change interventions

**DOI:** 10.1080/21642850.2023.2268684

**Published:** 2023-10-25

**Authors:** Rik Crutzen, Gjalt-Jorn Ygram Peters

**Affiliations:** a Department of Health Promotion, Maastricht University/CAPHRI, Maastricht, The Netherlands; b Department of Theory, Methods & Statistics, Faculty of Psychology, Open University of the Netherlands, Heerlen, The Netherlands; c Faculty of Psychology and Neuroscience, Department of Work and Social Psychology, Maastricht University, Maastricht, The Netherlands

**Keywords:** Determinants, predictors, regression, multivariate, bias

## Abstract

**Objective:**

Regression analyses are commonly used for selecting determinants to target in behavior change interventions, but the aim of this article is to explain why regression analyses are not suitable for this purpose (i.e. the regression trap).

**Methods:**

This aim is achieved by providing (1) a theoretical rationale based on overlap among determinants; (2) a mathematical rationale based on the formulas that are used to calculate regression coefficients; and (3) examples based on real-world data.

**Results:**

First, the meaning of regression coefficients is commonly explained as expressing the association between a determinant and a target behavior ‘holding all other predictors constant.’ We explain that this often boils down to ‘neglecting a part of the psyche.’ Second, we demonstrate that the interpretation of regression coefficients is distorted by correlations between determinants. Third, the examples provided demonstrate the impact this has in practice. This results in interventions targeting determinants that are less relevant and, thereby, have less impact on behavior change.

**Conclusion:**

There are theoretical, mathematical, and practical reasons why regression analyses, and by extension multivariate analyses relying on correlations, are not suitable to select determinants to target in behavior change interventions. Instead, intervention developers should consider univariate distributions and bivariate association estimates simultaneously and there are freely accessible tools available to do so.

An important step when developing behavior change interventions is to first identify determinants relevant to a target behavior. This is a key part of approaches to systematically develop interventions, such as Intervention Mapping (Bartholomew Eldredge et al., 2016), and is related to the mechanisms of an intervention as described in, for example, the Medical Research Council guidance regarding complex interventions (Skivington et al., 2021). In the context of this article, the term ‘determinants’ refers to psychological constructs that are postulated to (partly) cause behavior and that can be changed by means of behavior change interventions. Ideally, all determinants relevant to a specific behavior are targeted in an intervention to optimize its effectiveness. However, in practice, limited resources create a need to select those determinants that are *most* relevant to a specific behavior. Resources being finite concerns resources of both intervention developers and participants. This has an impact on the quantity and quality of intervention content that can be developed (e.g. because of budget constraints), but also delivered (e.g. in terms of participant burden). So, in practice, resources are *always* finite and there is a need to make a selection of determinants.

In order to make a selection of determinants to be targeted, it is important to know the associations between the determinants and the behavior of interest. The most straightforward association measure is the zero-order correlation coefficient, but regression analyses are more commonly used to make such selections, basing decisions on (significance of standardized) regression coefficients (β). In fact, it is common to start by reporting results of bivariate analyses (e.g. a correlation matrix of determinants), followed by reporting results of regression analyses as (a carefully built) ‘final model.’ For example, a meta-analysis of studies using the Reasoned Action Approach to understand health behaviors concluded – based on regression analyses – that ‘autonomy, instrumental attitude, and injunctive norm were not significant predictors of behavior’ (McEachan et al., 2016, p. 603). The results of such regression analyses are useful, because they provide insight into the proportion of variance (*R*^2^) in the outcome of interest (e.g. behavior) that can be explained by the predictors included in the model (e.g. determinants) (Peters & Crutzen, 2018).^1^ However, researchers (including ourselves) also frequently base conclusions regarding selection of determinants to be targeted in future interventions on such results (e.g. Beaujean et al., 2013; Ten Hoor et al., 2013). Such conclusions can have far-reaching consequences in the context of determinant selection. This context of determinant selection differs from the context of variable selection as discussed by, for example, Chowdhury and Turin (2020). In the latter, variable selection means choosing among many variables in a dataset which ones to include in a final model and finding a balance between parsimony of the model and its accuracy in terms of prediction. In the context of selecting determinants, the aim is not to build a model but instead to select those determinants that are most relevant to target among all (potentially) relevant determinants.

The aim of this article is to explain why regression analyses^2^ are not suitable for selecting determinants to target in behavior change interventions (i.e. the regression trap). To achieve this aim, we first provide a theoretical rationale based on overlap among determinants. This overlap is grounded in theories on behavior^3^ and appears to reflect fundamental characteristics of psychology. Second, we provide a mathematical rationale based on the way in which regressions coefficients are calculated. This in order to demonstrate that the interpretation of regression coefficients is distorted by correlations between determinants. Third, using real-world data from previously conducted studies, we provide examples on the impact this has in practice and discuss a possible solution.

## Theoretical rationale

A convenient feature of regression analyses is that overlap among predictors in their explanation of the outcome of interest is removed from the equation (quite literally, as demonstrated in the next section providing a mathematical rationale). This correction of overlap is very useful as it enables better estimation of the variance in the outcome that is explained by *all* predictors included in the model together. However, this overlap among predictors is in itself highly problematic when dealing with the separate regression coefficients of psychological constructs as predictors (Azen & Budescu, 2003; Budescu, 1993; Elwert & Winship, 2014). The meaning of regression coefficients is commonly explained as expressing the association between the predictor and the outcome *holding all other predictors constant*. This happens by dividing up the total explained variance among the predictors. If two predictors explain the same variance in the dependent variable, some of that explained variance is assigned to one predictor, and the rest to the other.

If two predictors partly overlap in their definition, or, in other words, if the definitions of the psychological constructs represented by the two predictors contain overlapping aspects of the psyche, then the shared explained variance belongs to both predictors simultaneously. For example, the Exercise Self-Regulation Questionnaire is used to measure motivation for exercise in line with the definitions of the Self-Determination Theory (Center for Self-Determination Theory, 2021). Identified regulation and intrinsic motivation are two types of motivation within this theory. Inspection of the measurement instruments for both constructs shows that ‘Because I believe exercise helps me feel better’ is used to measure identified regulation and that ‘Because it is interesting to see my own improvement’ is used to measure intrinsic motivation. Feeling better could be part of the improvement that a person sees. A second example in the same theory concerns the item ‘Because it feels important to me personally to accomplish this goal’, which is used to measure identified regulation, and the item ‘Because it is a challenge to accomplish my goal’, which is used to measure intrinsic motivation. Both items capture aspects of goal accomplishment. Assuming these items are valid, that means that identified regulation and intrinsic motivation partly overlap in their conceptual definitions: both constructs capture overlapping parts of the psyche. This makes sense because both are part of the overarching construct ‘motivation.’ However, the instructions of the Exercise Self-Regulation Questionnaire explicitly state that ‘You can use the individual subscale scores in your analyses.’ Using individual subscale scores in regression analyses would mean that if the variance in an outcome of interest is not solely assigned to, for example, identified regulation, the associated regression coefficient for identified regulation is biased. This is the case because the regression coefficient pertains to a unique part of identified regulation as well as an unknown subset of the part of identified regulation that overlaps with intrinsic motivation. In other words, *holding all other predictors constant* often means *neglecting a part of the psyche*: parts that are important as they constitute the constructs of interest. Assuming the construct’s measurement was valid, removing part of its variance invalidates it. When interested in intrinsic motivation, for example, and holding identified regulation constant, this would mean that part of intrinsic motivation is neglected as well because it is omitted from intrinsic motivation’s regression coefficient. This means that in such cases, the resulting regression coefficients no longer fully pertain to the determinants of interest, and as such, are not helpful to select determinants when developing behavior change interventions (Peters & Crutzen, 2018).

Once aware of this problem, it is easy to identify when predictors clearly represent overlapping determinants (i.e. constructs with measurement instruments that capture overlapping aspects of the psyche). More insidious are situations where careful inspection of the items in the constructs’ measurement instruments and their construct definitions do not readily reveal overlap. Unfortunately, the measurement of two constructs can rely on similar aspects of the psyche without obvious indicators.

Current conceptualizations of determinants of behavior are indicative of this ubiquitous overlap of psychological constructs. For example, in their conceptualization of affective determinants of health behaviors, Williams et al. (2019, p. 1267) notice that ‘these affective factors overlap in many ways with the cognitive factors that have been a focus of health behavior research for over 60 years’ (e.g. anticipated responses to exercise or drug use^4^). Another example are conscious and unconscious determinants of behavior, which ‘can be defined as two distinct but overlapping systems of learning and memory that explain and predict human decision-making, thoughts, and behaviors’ (Vrabel & Zeigler-Hill, 2020). The latter illustrates how overlap among determinants is not only an issue of definition, but actually reflects how the psyche works (Peters & Crutzen, 2017). Not only do determinants across various theories overlap, but determinants within each theory often overlap as well.

The problem this overlap causes for selecting determinants has already been acknowledged in the discussions on the predecessors of the Reasoned Action Approach long ago: ‘Because each component's measures appear to incorporate both attitudinal and normative influences, the relative importance of attitudinal and normative considerations for a particular behavior may well be distorted’ (Miniard & Cohen, 1979, p. 109). Given that overlap among psychological constructs typically seems to be the norm rather than the exception, that same distortion generalizes as well. In addition, this does not only concern measurement of constructs, but also manipulation as acknowledged by the theorists themselves: ‘it is impossible to tell, in advance, the exact extent to which a given manipulation or a given item of information will influence a person’s attitude or subjective norm’ (Fishbein & Ajzen, 1981, p. 344).

In short, overlap among determinants is grounded in theories on behavior and appears to reflect fundamental characteristics of psychology. This conceptual overlap manifests in correlations between determinants, and correlation matrices in studies on determinants show that these correlations are mostly non-trivial. This is in line with the crud factor, which refers to ‘the epistemological concept that, in correlational research, all variables are connected through causal structures which result in real non-zero correlations between all variables in any given dataset’ (Orben & Lakens, 2020, p. 241). In fact, correlations between determinants are part and parcel of theories on behavior as indicated by an ontology-based modeling system^5^ applied to 76 theories (Hale et al., 2020). This is also key to the mathematical rationale of why using regression coefficients is problematic in determinant selection.

## Mathematical rationale

Regression coefficients are calculated from zero-order correlation coefficients. For demonstration purposes, we use the simplest case when selecting determinants: a regression model in which the target behavior (y) is regressed on two determinants ($x_{1}$ and $x_{2}$). The conclusion is the same regardless of whether two or more determinants are used as predictors in the model: correlations between determinants distort the interpretation of regression coefficients in the context of selecting determinants (note that this problem is different from the problem of multicollinearity, which simply inflates coefficients’ error variances without necessarily introducing bias^6^). Using an example with only two determinants facilitates comprehension of the formula used to calculate regression coefficients and the visualization of the impact that correlation between determinants has on these regression coefficients.

The formula used to calculate regression coefficients contains the correlation between the target behavior and the first determinant ($r_{x_{1}y}$); the target behavior and the second determinant ($r_{x_{2}y}$); and the correlation between both determinants ($r_{x_{1}x_{2}}$) (Keith, 2019). The formula to calculate the regression coefficient of the first determinant (${\text{β}}_{x_{1}}\;$) is:

$${\text{β}}_{x_{1}}=\frac{r_{x_{1}y}-(r_{x_{2}y\cdot }r_{x_{1}x_{2}})}{1-r_{x_{1}x_{2}}}$$

The formula to calculate the regression coefficient of the second determinant (${\text{β}}_{x_{2}}$) is similar; only exchanging $r_{x_{1}y}$and $r_{x_{2}y}$. The key is that the correlation between both determinants is used to calculate both regression coefficients. A visualization based on this formula is helpful to gain insight into the impact this correlation between determinants has on regression coefficients. There is a Shiny application available (Brown, 2021) to create such visualizations based on specified values of the correlations between the target behavior and the first and second determinant respectively.

**Figure 1.**
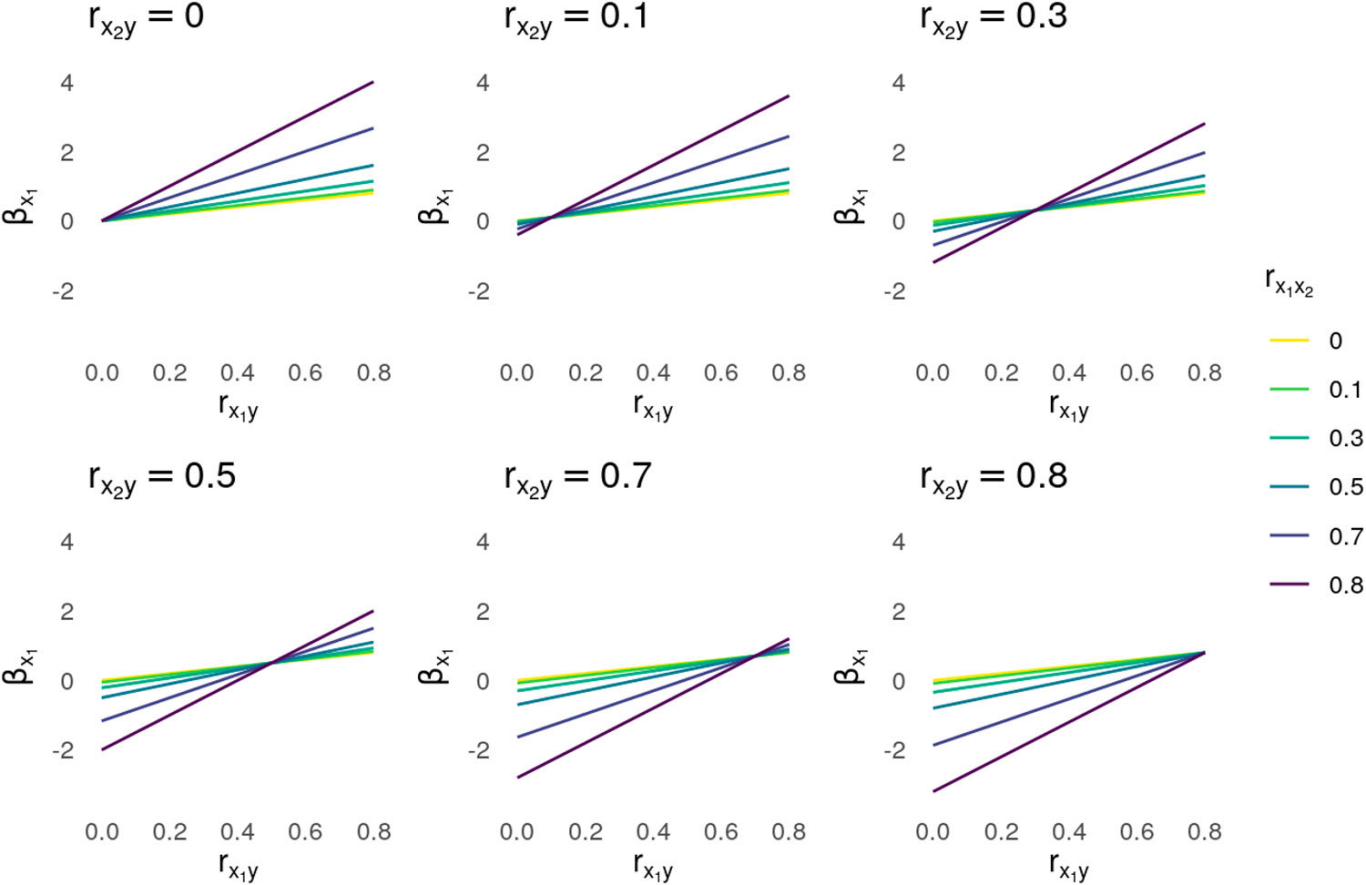
Impact of correlation between determinants on regression coefficients. Note: These panels show the relationship between $r_{x_{1}y}$ (*x*-axis) and ${\text{β}}_{x_{1}}$ (*y*-axis) for six values of $r_{x_{2}y}$ (in the different panels) and six values of $r_{x_{1}x_{2}}$ (represented by different lines). The code that produces this figure is available at https://osf.io/q9scj/.

Figure 1 visualizes the relationship between $r_{x_{1}y}$(x-axis) and ${\text{β}}_{x_{1}}$(*y*-axis) for six values of $r_{x_{2}y}$ (in the different panels) and six values of $r_{x_{1}x_{2}}$ (represented by separate lines): 0 (independence), .1 (a weak association), .3 (a moderate association), .5 (a strong association), .7 (about 50% overlap), and .8 (about two-thirds overlap) (Rosenthal, 1996). These values are used in the formula above to calculate ${\text{β}}_{\;x_{1}}$and subsequently visualized by means of R. When there is no correlation between determinants ($r_{x_{1}x_{2}}$ =  0), it can be derived from the formula above that the regression coefficient of the first determinant is equivalent to the correlation between the first determinant and the target behavior:

$${\text{β}}_{x_{1}}=\frac{r_{x_{1}y}-(r_{x_{2}y\cdot }0)}{1-0}=r_{x_{1}y}$$

This can be seen in Figure 1 by the yellow line representing completely independent predictors ($r_{x_{1}x_{2}}=0$) being a perfect diagonal where for each data point, the value on the x-axis is equal to the value on the y-axis (i.e. the identity line).

Deviations from this identity line are indicative of the interpretation of regression coefficients being distorted by correlations between determinants. In general, the stronger this correlation between determinants is, the more the value of the regression coefficient deviates from the identity line, with the darkest line in purple, corresponding to an association of ($r_{x_{1}x_{2}}=.8$), showing the highest potential for bias. Furthermore, the panels in Figure 1 demonstrate that this bias depends on the correlation between the second determinant and the target behavior. The value of the regression coefficient can both be lower and higher than the correlation with the target behavior, with intersecting lines (in Figure 1) when the associations for both predictors with the outcome of interest are equal (i.e. $r_{x_{1}y}=r_{x_{2}y}$).

So, the correlation between determinants has an impact on regression coefficients. While such regression coefficients might give an estimate of the ‘unique’ contribution of a determinant, this only concerns unique in the sense of the contribution not being shared with other predictors in the regression model (Johnson, 2000). For this contribution to represent the unique contribution of a predictor in the natural world, the formula used to compute the regression coefficients from the correlation coefficients would have to incorporate a way to calculate this unique contribution, since it cannot be inferred from the correlation coefficients alone. This makes comparison of regression coefficients inappropriate if the goal is to select those determinants *most* relevant to a specific behavior. In the next section, we illustrate the impact this has in practice using examples based on real-world data.

## Practical examples

The key point of the examples provided below is that selections based on comparing regression coefficients can lead to targeting determinants that are *less* relevant in comparison to other determinants. Targeting determinants that are less relevant to a target behavior results in less effective behavior change interventions. The examples provided are based on the Reasoned Action Approach and its predecessors (Fishbein & Ajzen, 2010). The advantage of using examples based on the Reasoned Action Approach is that intention to engage in a behavior is regressed on the same determinants in all studies: attitude, perceived norm, and perceived behavioral control. However, the key point is independent of the theory used and applies to all situations in which selections are made regarding determinants to be targeted in an intervention. Table 1 provides correlations and regression coefficients of determinants in previous studies focusing on various topics. The examples are taken from the book by Fishbein and Ajzen (2010) and all data presented in this table are available in the publications cited in this table.

**Table 1.** Examples of correlations and regression coefficients based on real-world data.

Topic	Attitude		Perceived norm		Perceived behavioral control	
	$r_{x_{1}y}$	${\text{β}}_{x_{1}}$	$r_{2y}$	${\text{β}}_{x_{2}}$	$r_{x_{3}y}$	${\text{β}}_{x_{3}}$
Physical activity (Armitage, 2005)	0.37	0.12	0.35	0.19	0.65	0.57
Taking ecstasy (Orbell et al., 2001)	0.75	0.44	0.69	0.24	0.34	0.58
Driving after drinking alcohol (Armitage et al., 2002)	0.71	0.34	0.71	0.41	0.64	0.23

Imagine an intervention developer who wants to select those determinants that are *most* relevant based on the findings presented in Table 1. In the first example, focused on physical activity, the values of both the correlation and the regression coefficient are the highest for perceived behavioral control in comparison to the other two determinants. In this example, perceived behavioral control would be the first determinant to be selected. With regard to the second determinant to be selected, the value of the regression coefficient for perceived norm is higher than the regression coefficient for attitude, but the value of the correlation for attitude is slightly higher. So, in a situation where resources are limited, attitude is a more relevant determinant to intervene upon based on the values of the correlations for perceived norm and attitude (because – in contrast to regression coefficients – these values are not distorted by correlations between determinants).^7^ In the second example, focused on taking ecstasy, the value of the regression coefficient for perceived behavioral control is higher in comparison with the values of the regression coefficients for the other determinants. However, the values of correlations for both attitude and perceived norm are higher then for perceived behavioral control. Hence, these determinants are both more relevant to intervene upon in comparison to perceived behavioral control, despite that the value of their regression coefficients is lower. In the third example, focused on driving after drinking alcohol, the values of the correlations for attitude and perceived norm are the same. The values of the regression coefficients for these determinants, however, give the impression that perceived norm is a more relevant determinant to intervene upon in comparison to attitude.

In sum, these examples demonstrate that making selections based on regression coefficients, instead of on bivariate associations such as the zero-order correlation, might lead to targeting determinants that are less strongly correlated with the outcome of interest. This results in interventions targeting determinants might be less relevant (because the regression coefficients are distorted) and, thereby, have less impact on behavior change.

## Discussion

This article explains why regression analyses are not suitable for selecting determinants to target in behavior change interventions. While regression analyses were used for demonstration purposes, this conclusion holds for multivariate^8^ analyses more broadly. Also more advanced analyses, such as structural equation modeling, are often not suitable for selecting determinants as they rely on correlations between determinants (Hamilton et al., 2020). Furthermore, the theoretical rationale strengthens this conclusion as overlap among determinants is grounded in theories on behavior and reflects how the psyche works: correlated determinants seem inevitable. So, bivariate analyses regarding associations with the outcome of interest are needed when selecting determinants, because these are not distorted by overlap among determinants and reflect the actual association between determinants and outcome of interest.

To circumvent the regression trap, bivariate analyses need to be combined with insight into univariate distributions of determinants to make selection based on relevance (Crutzen et al., 2017). Bivariate analyses can provide insight into the associations between multiple determinants and the target behavior. This is needed because a determinant has to be associated with behavior to be selected. These bivariate analyses need to be combined with the univariate distribution of each determinant. If a determinant is associated with behavior, but most people already have the desired value (e.g. they hold a positive attitude towards the behavior), for example, then this determinant may be forgone as an intervention target or may merely be reinforced in an intervention. However, if most people do not have the desired value yet, then this determinant would be a more viable intervention target as there is more room for improvement. Confidence-Interval Based Estimation of Relevance (CIBER) combines both bivariate analyses and univariate distributions into a visualization that facilitates selection of determinants based on relevance (Crutzen et al., 2017). These insights regarding relevance need to be combined with changeability to make final decisions in terms of intervention targets (Bartholomew Eldredge et al., 2016). When making such decisions, triangulation of insights from, for example, systematic reviews and qualitative research can lead to a better understanding that is helpful to guide these decisions (Ruiter & Crutzen, 2020).

We have deliberately used the term ‘associations’ in the context of selecting relevant determinants. Although the term ‘determinants’ implies causality, neither multivariate analyses nor bivariate analyses are on their own informative regarding the causality of determinants. Rohrer et al. (2022), for example, provide a concise summary of how causal-inference problems also affect path models. The type of conclusions that can be drawn depends on the study design that is used, not solely the model that is used for analyses. However, the superiority of bivariate analyses over multivariate analyses for selecting determinants is independent of whether a cross-sectional, longitudinal or experimental design is used. In fact, limited experimental data allowing for causal conclusions on (what causes) changes in determinants is a ‘known unknown’ in behavior change (Hagger et al., 2020). More recently, attention has been drawn to a complex adaptive systems approach to behavior change research, which acknowledges the interconnectedness between constructs as one of its features (Heino et al., 2021).

Even if correlations between predictors in their explanation of the outcome of interest can unequivocally be attributed to their causal structure and constructs capturing overlapping aspects of the psyche can be ruled out as an explanation, there is an additional reason to avoid using regression analyses for selection of intervention targets. This concerns the ethics and feasibility of intervening implied by the explanation of the meaning of regression coefficients as expressing the association between the predictor and the outcome *holding all other predictors constant*. This concern is not limited to psychological constructs that can be changed by means of behavior change interventions. For example, a previous study focused on the effects of smoking level, HIV status, and age on stroke. The interpretation of the effect of age in this study requires ‘holding a person’s smoking level and HIV status constant as they age, which would be unethical for smokers (among whom reduction should be encouraged) and infeasible even if desirable for those HIV negative (because some will maintain unsafe practices)’ (Westreich & Greenland, 2013, p. 297). This concern regarding the ethics and feasibility of intervening applies to psychological constructs as well. For example, it would be both undesirable and impractical when stimulating a positive attitude change to instruct intervention participants to hold their perceived behavioral control – or any other psychological construct for that matter – constant. Quite the opposite: interventions generally target multiple determinants, rendering such a scenario is impossible.

In sum, there are theoretical, mathematical, and practical reasons why regression analyses, and by extension multivariate analyses relying on correlations, are not suitable to select determinants to target in behavior change interventions. Instead, intervention developers should consider univariate distributions and bivariate association estimates simultaneously and there are freely accessible tools available to do so (Crutzen et al., 2017; Peters, 2021).

## Data Availability

Data sharing not applicable – no new data generated.

## Notes

1 Although proportion of variance explained has been described as a pessimistic measure (Sutton, 1998).

2 While the examples used in this article concern linear regression models, the same reasoning applies to other regression models (e.g., logistic regression models).

3 A theory is a set of concepts and/or statements with specification of how phenomena relate to each other. Theory provides an organizing description of a system that accounts for what is known, and explains and predicts phenomena (Davis et al., 2015).

4 For example, somebody who likes running may *enjoy* experiencing a runner’s high (an affective determinant of behavior), but may also consider *beneficial health effects* of running to be optimal when running for at least the period of time that also yields that runner’s high (a cognitive determinant of behavior). In this case, the person’s causal representations around runner’s high are an intrinsic part of both constructs.

5 A system for modeling theories of behavior using a formal representation of the constructs within theories and the ways in which constructs relate to or interact with each other (Hale et al., 2020).

6 Multicollinearity refers to the phenomenon that if predictors correlate, each predictor supplies less information than if they had been orthogonal. This multicollinearity (as expressed by a predictor’s tolerance, i.e. 1 minus the *R*^2^ obtained when regressing the predictor on all other predictors, e.g. if the *R*^2^ of attitude, predicted by perceived norm and behavioral control, is .67, then attitude’s tolerance is .33) then causes the standard error of the predictor to become larger (by a factor equal to the variance inflation factor [VIF], which is the reciprocal of its tolerance; if attitude’s tolerance is .33, then its VIF is 3 [i.e., 1/.33] and its standard error is three times larger than if its correlation with perceived norms and behavioral control had been 0). Multicollinearity is a problem because it results in very unstable coefficient estimates (as manifesting in, for example, wide confidence intervals). However, the degree to which the predictors are unbiased estimates of the population value is not influenced. This in contrast to the problem we outline in this article, which does constitute bias in regression coefficients.

7 In principle, using a frequentist approach, decisions following from comparison of correlations should take the confidence intervals of these correlation coefficients into account, because they are indicative of the accuracy with which these correlations can be estimated. The wider these confidence intervals are, the more likely it is that there is overlap among the confidence intervals of various determinants, which makes it harder to differentiate among determinants. This stresses the importance of taking desired accuracy of parameter estimates into account during study planning (Peters & Crutzen, 2021).

8 The term multivariate, as opposed to multivariable, is used in contrast to bivariate in order to distinguish analyses including more than two variables with those including only two variables (Denis, 2020).

## Open Scholarship

This article has earned the Center for Open Science badges for Open Materials. The materials are openly accessible at https://osf.io/q9scj/.
